# Glucose monitoring during hemodiafiltration: a comparison of continuous, capillary, and dialysis-line measurements

**DOI:** 10.1093/ckj/sfag142

**Published:** 2026-05-05

**Authors:** Marcia S Carvalho, Christiane A Kojima, Erica P Rocha, Daniel M Costa, Andrea O Magalhães, Cynthia de M Borges, Eduarda Vitoria S Rocha, Andreia S Fernandes, Larissa Lorena G Resende, Amanda Leal, Luciane Alves, Lilian Cuppari, Rosilene M Elias

**Affiliations:** Nefrostar Kidney Care, Sao Paulo, Brazil; Splendore Kidney Care, Sao Paulo, Brazil; Nefrostar Kidney Care, Sao Paulo, Brazil; Nefrostar Kidney Care, Sao Paulo, Brazil; Nefrostar Kidney Care, Sao Paulo, Brazil; Nefrostar Kidney Care, Sao Paulo, Brazil; Nefrostar Kidney Care, Sao Paulo, Brazil; Nefrostar Kidney Care, Sao Paulo, Brazil; Nefrostar Kidney Care, Sao Paulo, Brazil; Nefrostar Kidney Care, Sao Paulo, Brazil; Nefrostar Kidney Care, Sao Paulo, Brazil; Nefrostar Kidney Care, Sao Paulo, Brazil; Nefrostar Kidney Care, Sao Paulo, Brazil; Universidade Federal de Sao Paulo, Department of Medicine, Nephrology, Sao Paulo, Brazil; Nefrostar Kidney Care, Sao Paulo, Brazil; Splendore Kidney Care, Sao Paulo, Brazil; Universidade de Sao Paulo, Department of Medicine, Service of Nephrology, Sao Paulo, Brazil; Universidade Nove de Julho, Postgradruate program, Sao Paulo, Brazil

To the Editor,

Diabetes mellitus is the leading cause of chronic kidney disease (CKD) worldwide [1, 2]. Glycemic control in these patients is a major clinical challenge, particularly in those with advanced disease, significant alterations occur in the pharmacokinetics of glucose-lowering agents [3]. It has been recommended to maintain glycated hemoglobin (HbA1c) levels between 7.5% and 8.5%, with strict control of both fasting and postprandial glucose, while minimizing hypoglycemic events.

Glycemic control in dialysis patients is influenced by multiple factors, including the glucose content of the dialysate, food intake during sessions, and adjustments in hypoglycemic therapy. Therefore, it is essential to evaluate glycemic control on both dialysis and nondialysis days.

For glucose monitoring, there are no specific recommendations distinguishing community-based patients from those on hemodialysis. Patients are therefore instructed to measure capillary blood glucose, and physicians adjust medication regimens according to periods of better or poorer control. During hemodialysis sessions, glucose measurement is performed according to medical prescription and individualized for each patient. Hypoglycemia during sessions is not uncommon and is promptly managed with intravenous glucose supplementation. HbA1c remains the gold standard for assessing glycemic control although it does not capture glucose variability or hypoglycemic episodes [4]. In this context, continuous glucose monitoring (CGM) has gained increasing importance, as it allows a more detailed and individualized evaluation of glycemic profiles.

In the current study we aimed to evaluate glycemic measurements in patients with diabetes on maintenance hemodiafiltration from CGM, capillary blood, and the dialysis circuit line.

This is a cross-sectional analysis of glucose monitoring in adult patients with diabetes undergoing maintenance hemodiafiltration. Patients were recruited from four dialysis centers in the period between 1 May 2024 and 1 December 2025.

Inclusion criteria were adult patients with diabetes mellitus type 1 or type 2, with CKD, stable on maintenance hemodiafiltration for at least 3 months, using CGM. All patients were followed by the same endocrinologist who confirmed the diagnosis and prescribed hypoglycemic medications.

The main outcome was the glucose measurement obtained from three different settings: (i) CGM, (ii) finger capillary sampling, and (iii) dialysis line (before dialysis, and 1 and 2 h after the procedure have started). Glucose was measured in the same laboratory using hexokinase. CGM was performed using a flash glucose monitoring system (FreeStyle Libre, Abbott Diabetes Care, USA). Capillary blood glucose was measured using a portable glucometer based on an enzymatic electrochemical method.

The concentration of glucose in the dialysis solution was identical for all patients. Comparisons among the three measurement methods were performed using analysis of variance or alternative Kruskall–Wallis, with post-test when significant.

**Figure 1:**
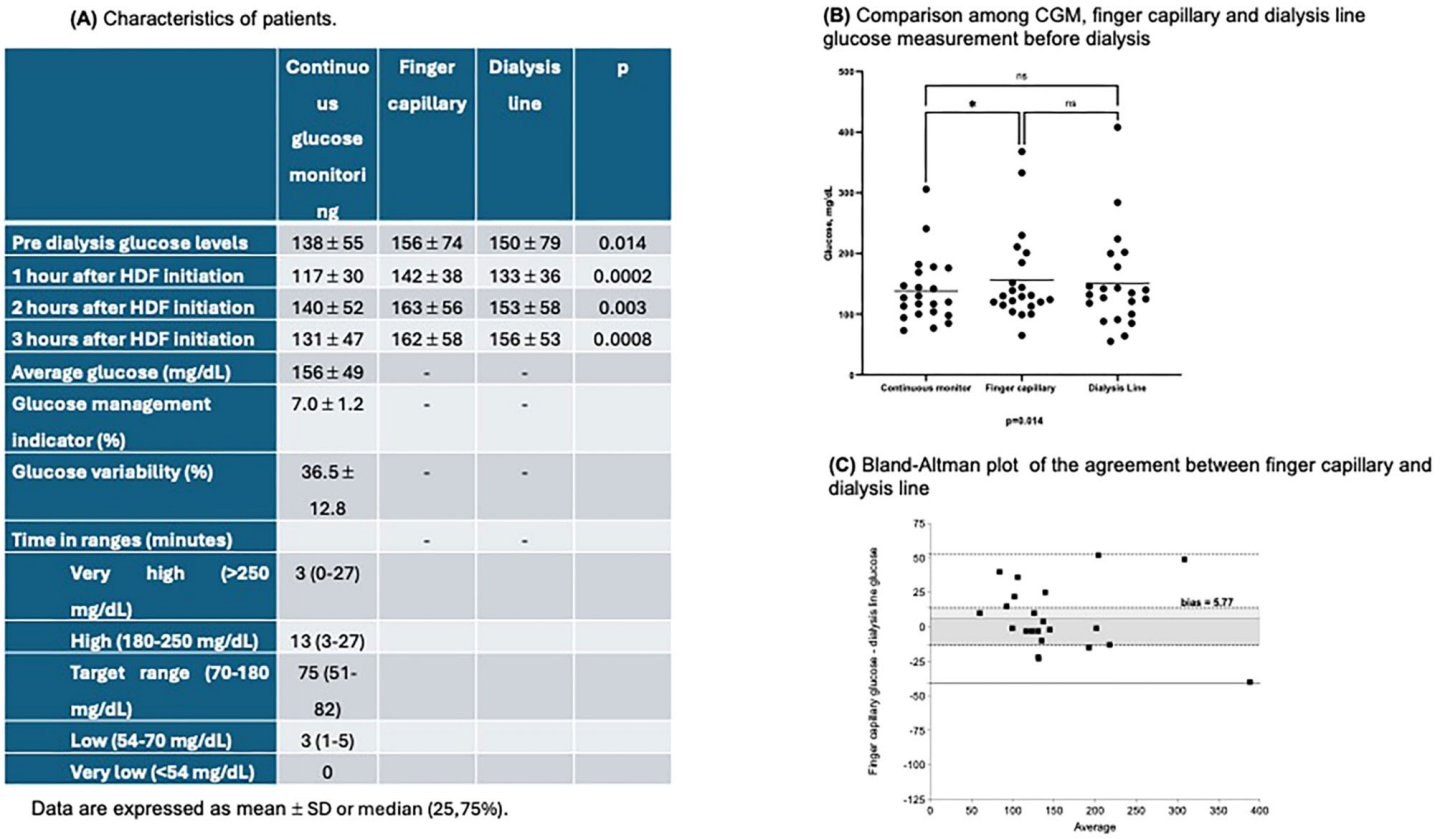
(A) Characteristics of patients. (B) Comparison among CGM, finger capillary, and dialysis line glucose measurement before dialysis. (C) Bland–Altman plot of the agreement between finger capillary and dialysis line.

We enrolled 66 glucose measurements in 22 patients. Blood glucose values obtained from CGM, finger capillary sampling, and dialysis line before dialysis were summarized in Fig. 1A. Glucose levels obtained from CGM were lower in all comparisons, with no significant difference between finger capillary sampling and dialysis line (Fig. 1B). Glucose levels were slightly higher—an average of 23 mg/dl—in finger capillary than in dialysis line in 10 patients (45.4%), and lower—an average of 10.5 mg/dl—in 12 patients (54.6%). The Bland–Altman plot revealed consistently lower values from CGM compared to finger capillary sampling, with an agreement between finger capillary and dialysis line illustrated in Fig. 1C (bias 5.8, limits of agreement from −41.0 to 52.5).

CGM systematically underestimates glucose levels compared with capillary and dialysis line measurements in patients undergoing hemodiafiltration. Lower CGM readings likely result from interstitial–capillary differences and temporal lag relative to capillary and dialysis line measurements. Capillary and dialysis line measurements show good agreement, supporting their use as interchangeable methods in clinical practice.
